# Effects of polyvinyl chloride microplastics with different particle sizes on growth, physiology, and intestinal microbiota of *Macrobrachium rosenbergii*

**DOI:** 10.3389/ftox.2026.1797231

**Published:** 2026-04-10

**Authors:** Qiaoyan Zhou, Yakun Wang, Jie Wei, Tianhui Jiao, Sikai Xu, Kunhao Hong, Yayi Huang, Zikang Tu, Yurong Zhang, Yongchun Huang, Lingyun Yu

**Affiliations:** 1 College of Fisheries, Jimei University, Xiamen, China; 2 Key Laboratory of Tropical and Subtropical Fishery Resource Application and Cultivation of Ministry of Agriculture and Rural Affairs, Pearl River Fisheries Research Institute, Chinese Academy of Fishery Sciences, Guangzhou, Guangdong, China; 3 College of Biological and Environmental Sciences, Zhejiang Wanli University, Ningbo, China

**Keywords:** growth and development, gut microbiota, Macrobrachium rosenbergii, polyvinyl chloride microplastics, toxicity

## Abstract

**Background:**

This study addresses a critical knowledge gap regarding the long-term, multi-size-dependent toxic effects of polyvinyl chloride microplastics (PVC-MPs) on economically important freshwater aquaculture species, specifically the giant freshwater prawn (Macrobrachium rosenbergii). Given the severe microplastic pollution in intensive aquaculture regions like China’s Pearl River Delta, understanding these impacts is vital for ecological risk assessment and sustainable aquaculture.

**Methods:**

Post-larval M. rosenbergii were exposed to environmentally relevant concentrations (1 mg/L) of fluorescent PVC-MPs of three particle sizes (30, 60, 90 μm) for 28 days, followed by a 14‐day recovery period in clean water. A comprehensive analysis was conducted, including assessments of growth and survival, microplastic accumulation, tissue ultrastructure and apoptosis, gene expression related to antioxidant defense, immunity, and growth, and intestinal microbiota composition.

**Results:**

Exposure to PVC-MPs significantly inhibited growth and reduced survival, with the 90 μm particles showing the strongest effect. MPs accumulated in gills and intestines within one day and entered the circulatory system. They caused significant ultrastructural damage and increased apoptosis in gills, intestines, and hepatopancreas. Molecular responses showed an initial upregulation followed by suppression of antioxidant and immune-related genes after long-term exposure. Gut microbiota analysis revealed dysbiosis, characterized by decreased Firmicutes and increased Proteobacteria. Recovery experiments indicated particle-size-dependent clearance: large (90 μm) MPs were completely eliminated within 14 days, while smaller particles (30, 60 μm) persisted in tissues.

**Conclusion:**

PVC-MPs impair M. rosenbergii growth through physical damage, oxidative stress, immune suppression, and gut microbiota dysbiosis, with toxicity and clearance efficiency being particle-size-dependent. These findings provide insights into the ecological risks of microplastics in aquaculture.

## 1 Introduction

Plastics, due to their low cost, lightweight, and high durability, have become one of the most widely used synthetic materials worldwide (Ghosh et al., 2023). However, its large-scale production and improper management after consumption have led to a large amount of plastic waste entering the natural environment (Organisation de coopération et de développement économiques, 2022). Currently, approximately 400 million tons of plastic waste are generated globally each year, and this figure is projected to continue rising (Lampitt et al., 2023; Borrelle et al., 2020). If current trends persist, environmental accumulation across rivers, lakes, and oceans could reach 12 billion tons by 2050 (Geyer et al., 2017). Under the influence of ultraviolet radiation and weathering, discarded plastics gradually break down into fragments with a diameter of less than 5 mm, which are collectively referred to as microplastics (Thompson et al., 2004). In recent years, microplastic pollution in aquatic environments has become a globally contentious issue. Microplastics have been detected in a wide range of aquatic habitats, including the deep-sea trenches, polar regions, and coastal wetlands (Deng et al., 2025; Chen et al., 2024; Yang et al., 2023; Dong et al., 2021). Compared with relatively systematic research on microplastics in marine environments (Tang et al., 2023; Biswas and Subodh, 2024), the distribution patterns and ecological risks of microplastics in freshwater ecosystems remain poorly understood and lack systematic investigation (Miloloža et al., 2020). In fact, approximately 80% of the aquatic plastic waste originates from land and is transported to the sea through freshwater systems such as rivers (Siegfried et al., 2017). Studies have shown that the abundance of microplastics in freshwater environments may even be higher than that in marine environments. For instance, the abundance of microplastics in sediments from the Yangtze River Basin can reach as high as 25 to 340 items per kilogram (Peng et al., 2017). The severity of microplastic pollution in China’s freshwater environments is significantly higher than that in marine environments: for example, the abundance of microplastics in the surface water of Taihu Lake ranges as high as 3,400 to 25,800 items per cubic meter (Fu et al., 2020), whereas in the more polluted coastal waters of the Bohai Sea and Yellow Sea, the microplastic abundance in surface water is generally less than 1 item per cubic meter (Fu et al., 2020). Mahamud et al. (2022) and Yu et al. (2023) also pointed out that the pollution of microplastics in freshwater aquaculture systems is particularly severe. Furthermore, the intake of microplastics can cause a variety of adverse effects on aquatic organisms, including oxidative stress, endocrine disruption, decreased feeding, stunted growth, genetic toxicity, and even death (Li Y. et al., 2025; Zhang T. et al., 2022).

**FIGURE 1:**
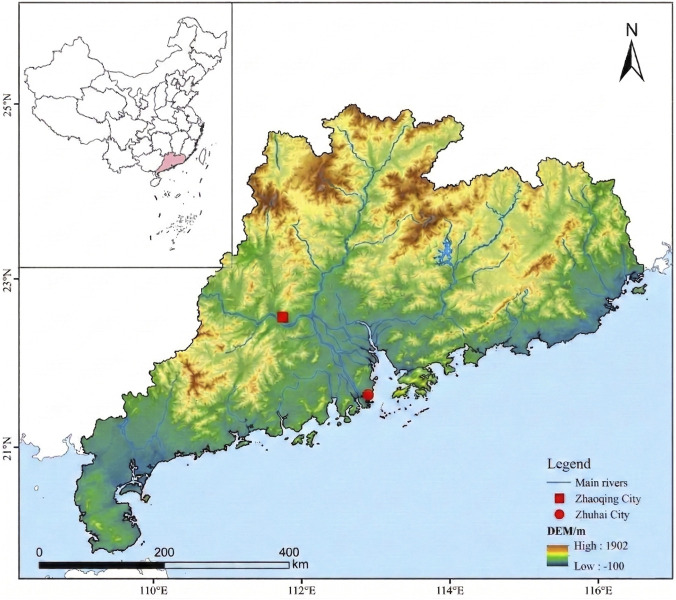
Sampling sites for microplastic water samples in M. rosenbergii aquaculture ponds in the Pearl River Delta (Zhuhai and Zhaoqing).

Prized for its mechanical strength, chemical resistance, and affordability, polyvinyl chloride (PVC) is ubiquitous across pipelines, medical supplies, and packaging industries (Safarpour et al., 2021). However, with nearly 60% of its global annual production ultimately entering municipal waste streams, PVC gradually fragments into polyvinyl chloride microplastics (PVC-MPs) (Liu et al., 2020; Lu et al., 2023; Han et al., 2024). Notably, PVC-MPs are now detected even more frequently than polystyrene across both freshwater and marine environments (Nava et al., 2023), with striking concentrations recorded in major inland water bodies such as China’s Poyang Lake (Jian et al., 2020). This contamination is particularly acute in the Pearl River Delta—a region where intensive aquaculture intersects with massive industrial plastic consumption. Through industrial discharge, domestic sewage, and wastewater treatment effluents, large volumes of microplastics (such as textile fibers) are transported via river networks directly into the estuary, where they inevitably accumulate in aquaculture ponds (Wang et al., 2025; Ma et al., 2020). Crucially, our preliminary investigations confirm that PVC-MPs ranging from 20 to 100 μm are already pervasive in local *Macrobrachium rosenbergii* farming systems (Supplementary Figure S1; Figure 1). This establishes that such pollutants have deeply infiltrated the rearing environments of this economically vital shrimp species in China.

*Macrobrachium rosenbergii* is a vital aquaculture species in China and Southeast Asia, valued for its rapid growth, high adaptability, and substantial economic value. In 2024, China’s domestic production reached 245,700 tons across approximately 700,000 mu, with Guangdong, Jiangsu, and Zhejiang provinces contributing over 90% of the total yield (Ministry of Agriculture and Rural Affairs of the People’s Republic of China, 2025). However, farmed *M. rosenbergii* are highly susceptible to microplastic pollution. Studies have reported microplastic concentrations up to 32.66 ± 5.10 items/g in their digestive tracts (Reunura and Prommi, 2022), which induces various adverse physiological effects (Jaikumar et al., 2021; Trinh et al., 2024). The severity of these impacts is strongly tied to particle size. For instance, Zhou et al. (2023) demonstrated that while the smallest polystyrene microplastics caused the most severe tissue accumulation and physical damage in shrimp, medium-sized particles triggered the greatest disruption to antioxidant enzyme activity. Furthermore, Kim et al. (2025) found that small polyethylene terephthalate particles inhibited growth and induced oxidative stress, whereas larger particles primarily altered gene expression and triggered a compensatory metabolic recovery. Consequently, microplastic toxicity is driven by complex, size-specific mechanisms rather than a simple inverse relationship with particle size.

However, current research on the particle size-dependent toxicity of microplastics still has notable limitations. Most existing studies focus on a narrow range of polymer types, primarily polystyrene and polyethylene terephthalate (Zhou et al., 2023; Kim et al., 2025); in contrast, PVC-MPs represent one of the most frequently detected polymer types in freshwater environments (Nava et al., 2023), yet the particle size-dependent toxic mechanisms of PVC-MPs remain largely unclear. Furthermore, the *Litopenaeus vannamei* is a marine/saline water aquaculture species, while the *M. rosenbergii*, as a typical freshwater economic shrimp species, may have different response mechanisms to microplastics due to differences in feeding behavior and the composition of its intestinal microbiota. Therefore, clarifying the specific physiological responses of *M. rosenbergii* to PVC-MPs of varying sizes is essential to accurately assess ecological risks in freshwater aquaculture.

To bridge this gap, we simulated typical aquaculture pond conditions in the Pearl River Delta. We exposed *M. rosenbergii* to an environmentally relevant concentration (1 mg/L) of PVC microplastics across three sizes (30, 60, and 90 μm). The study consisted of a 28-day exposure period followed by a 14-day depuration phase in clean water. To determine how PVC size affects the prawns, we evaluated their growth performance, histopathology, oxidative stress and immune responses, gut microbiota, and *in vivo* depuration rates. Based on current literature, we hypothesized the following: (1) Small microplastics easily penetrate tissues and enter circulation. Their low clearance rate leads to prolonged retention, causing chronic toxicity through sustained oxidative stress and apoptosis. (2) Large microplastics struggle to cross tissue barriers but cause acute physical blockages that impair feeding and respiration. However, because they remain largely in the digestive tract, the prawns can excrete them faster and show physiological recovery during the depuration phase. These findings will clarify the size-dependent mechanisms of microplastic toxicity in freshwater crustaceans, providing practical guidance for *M. rosenbergii* aquaculture and ecological risk assessments.

## 2 Materials and methods

### 2.1 Animal rearing and experimental design

The green fluorescent polyvinyl chloride microspheres used in the experiment were purchased from Big Goose Technology Co., Ltd. (Tianjin, China), with excitation and emission wavelengths of 488 nm and 518 nm, respectively. Characterization by transmission electron microscopy (TEM, Hitachi HT7800) and dynamic light scattering (DLS, Malvern Zetasizer Nano ZS) confirmed that the microspheres exhibited good monodispersity, uniform particle size, and satisfactory stability in freshwater systems, meeting the experimental requirements.

The post-larval *M*. *rosenbergii* used in the experiment were obtained from the Pearl River Branch of the National Freshwater Aquatic Germplasm Resource Bank, Pearl River Fisheries Research Institute, Chinese Academy of Fishery Sciences. The initial body weight and length of the post-larvae were 1.12 ± 0.54 g and 3.64 ± 0.58 cm, respectively. Prior to the experiment, they were acclimatized for 1 week in a recirculating aquaculture system with the water temperature maintained at 25 °C ± 2 °C, pH at 7.5 ± 0.2, and dissolved oxygen above 6.0 mg/L. They were fed daily to satiation with a commercial shrimp diet (crude protein ≥38%). Prior to the formal experiment, the animals were fasted for 24 h. A total of 800 healthy post-larval *M*. *rosenbergii* were selected in this study and randomly assigned to 16 glass aquaria with a size of 53 cm × 33 cm × 29 cm. The experiment was carried out simultaneously in parallel using a completely randomized block design, with 1 control group (Group C: no microplastic exposure) and 3 microplastic exposure groups (Groups E30, E60, and E90, exposed to 30 μm, 60 μm, and 90 μm microplastics, respectively). Each of the 4 treatment groups was set with 4 independent glass aquaria as independent biological replicates, and 50 post-larvae were stocked per tank (all indicator measurements were performed using juvenile shrimp randomly selected from the 4 replicates).

The exposure concentration of microplastics in all treatment groups was 1 mg/L. The exposure concentration used in this study was selected based on the commonly detected concentrations of microplastics in freshwater ecosystems (Shen, 2024; Han et al., 2020), and is consistent with those frequently employed in similar studies (Zhou et al., 2023; Duan et al., 2021; Zeng et al., 2023). The light-dark cycle was set as 12 h of light and 12 h of dark (light period from 08:00 to 20:00). During the experimental period, feed was provided daily at 9:00 and 17:00 at a rate of 5% of the total shrimp body weight. Residual feed and feces were removed 2 h after each feeding. One-third of the water volume was replaced with aerated tap water every 2 days to maintain stable water quality. The exposure period lasted for 28 days, followed by a 14-day recovery experiment.

### 2.2 Growth performance measurement

At 28 days of exposure, 30 post-larvae were randomly selected from each group. Their body length was measured using tpsDig2 software (F. James Rohlf, Stony Brook University, Stony Brook, NY, USA), and their body weight was measured using a Mettler Toledo AL-204 precision balance (Mettler Toledo, Inc., Shanghai, China). The Weight Gain Rate (WGR) and Specific Growth Rate (SGR) were calculated according to the method described by Liufu et al. (2025):

$$\text{Weight gain rate }\left(\text{WGR},\%\right)=\frac{Wt-Wo}{Wo}\times 100$$

$$\text{Specific growth rate }\left(\text{SGR},\%/d\right)=\frac{\ln Wt‐\ln Wo}{t}$$

where:

*Wo* and *Wt* represent the initial and final body weight (g), respectively;

*t* is the duration of the exposure period in days (28 days).

### 2.3 Microplastic distribution and residue analysis

On the first day of the experiment, 3 juvenile shrimp larvae were randomly selected from each experimental group and were first anesthetized using ice water. After the anesthesia was completed, the larvae were dissected and the gill tissues and intestinal tissues were completely separated. Subsequently, the tissue samples were rinsed three times with ultra-pure water to thoroughly remove the impurities adhering to the surface of the samples. Place the washed tissue samples flatly in the center of the slide, gently cover them with a cover glass, and finally use the Nikon Eclipse Ni-U fluorescence microscope to observe and record the distribution of microplastics in the gill and intestinal tissues under the excitation/emission wavelengths of 488/518 nm.

After 28 days of exposure, PVC-MPS in the hemolymph of juvenile shrimp were determined using solvent extraction coupled with pyrolysis-gas chromatography-mass spectrometry (Py-GCMS). Accurately weighed samples (to the nearest 0.001 g) were first dried to a constant weight at 60 °C. To remove organic matrices, the samples were sequentially washed three times with 30 mL of ethanol and n-hexane at 60 °C (10 min per wash, with the supernatant discarded each time). PVC was then extracted using 10 mL of chloroform under ultrasonication (40 kHz, 10 min), and this extraction process was repeated three times. The combined extracts were concentrated to approximately 1 g at 80 °C, transferred dropwise into a Py-GCMS sample cup, and heated until the solvent had completely evaporated. The samples were subsequently analyzed using a Py-GCMS system (a Frontier PY-3030D pyrolyzer interfaced with a Shimadzu GC-2030/QP2020NX). The samples were flash-pyrolyzed at 550 °C for 12 s, and the resulting pyrolyzates were separated on an Rtx-5MS capillary column (30 m × 0.25 mm × 0.25 μm). Helium was used as the carrier gas at a flow rate of 1.0 mL/min with a split ratio of 5:1. The oven temperature program was initiated at 40 °C (held for 2 min), ramped to 320 °C at a rate of 20 °C/min, and maintained for 14 min. The mass spectrometer was operated in full-scan mode (m/z 29–600) using an electron impact (EI) ionization energy of 70 eV. For data processing, naphthalene (retention time: ∼8.145 min, target ion: m/z 128) was selected as the characteristic pyrolysate indicator for the qualitative and quantitative analysis of PVC. A linear regression equation (y = 355783.4x-19082.34, R^2^ = 0.9991) was established using an external standard calibration method, with a limit of quantification (LOQ) of 0.060 μg per injection. After subtracting the background interference from procedural blanks, the final results were expressed as μg/g hemolymph (wet weight) (the GC-MS spectra for qualitative analysis are provided in Supplementary Figure S2).

At the conclusion of the 28-day PVC-MPs exposure period, surviving post-larvae from each experimental group were transferred to clean water free of microplastics for a 14-day recovery phase. Subsequently, the gills and intestinal tract of the post-larvae were dissected and collected. The residual status of PVC-MPs within these two tissue types was then observed and recorded using fluorescence microscopy.

### 2.4 Ultrastructure and apoptosis analysis

Tissues including gill, intestine, and hepatopancreas were collected from post-larvae at 14 days and 28 days of exposure, and after 14 days of recovery. The tissues were cut into 1 mm^3^ cubes and immediately fixed in pre-cooled 2.5% glutaraldehyde (prepared in phosphate buffer, pH 7.4) for 24 h, followed by fixation in 1% osmium tetroxide for 2 h. After fixation, the samples were dehydrated through a graded ethanol series (50%, 70%, 90%, 95%, 100%, 15 min each step) and cleared with propylene oxide, before being embedded in Epon 812 resin. Ultrathin sections (70 nm) were prepared using a Leica UCT25 ultramicrotome. The sections were double-stained with uranyl acetate and lead citrate, and observed under a Hitachi HT7800 transmission electron microscope to examine ultrastructural changes in subcellular organelles such as mitochondria and the endoplasmic reticulum.

Tissue samples (gill, intestine, and hepatopancreas) collected after 28 days of exposure were fixed in 4% paraformaldehyde for 24 h. Following dehydration through a graded ethanol series (75%, 85%, 95%, and 100%), clearing with xylene, and paraffin embedding, sections of 4 μm thickness were prepared using a Leica RM2016 microtome. The sections were digested with Proteinase K (37 °C, 20 min), and then incubated with TUNEL reaction mixture (TdT:dUTP:Buffer = 1:5:50, Promega, United States) in the dark at 37 °C for 1 h. Cell nuclei were counterstained with DAPI. Under UV excitation, nuclei of viable cells stained with DAPI exhibited blue fluorescence, whereas apoptotic nuclei positive for TUNEL displayed red fluorescence.

### 2.5 Analysis of related gene expression

Total RNA was extracted from hepatopancreas tissue using TRIzol reagent (Invitrogen, Waltham, MA, United States). The integrity and purity of the RNA were assessed by 1.0% agarose gel electrophoresis and NanoDrop 2000 spectrophotometry (A260/A280 ratio between 1.8 and 2.0). One microgram of RNA was reverse-transcribed into cDNA using M-MLV reverse transcriptase (Invitrogen). The expression of target genes (*CAT*, *SOD*, *TOLL*) was measured using a StepOnePlus Real-Time PCR System (Applied Biosystems, USA). The reaction mixture (20 μL) consisted of 10 μL SYBR Green Mix, 1 μL each of forward and reverse primers (10 μmol/L), 1 μL cDNA template, and 7 μL ddH_2_O. The thermal cycling protocol was as follows: initial denaturation at 95 °C for 3 min; followed by 35 cycles of denaturation at 95 °C for 40 s, annealing at 60 °C for 40 s, and extension at 72 °C for 30 s. The *β-actin* gene was used as an internal reference, and relative gene expression levels were calculated using the 2^−ΔΔCT^ method. Primer sequences for the target genes and the reference gene are listed in Table 1.

**TABLE 1:** Nucleotide sequences and sources of primers used for qRT-PCR.

Genes	Sequence (5′-3′)	Reference or Query Gene ID
*β-ACTIN* F	CAG​GGA​AAA​GAT​GAC​CCA​GA	AY626840
*β-ACTIN* R	GGA​AGT​GCA​TAC​CCC​TCG​TA	​
*CAT* F	ACT​TCA​TTA​CCC​TGA​GAC​CCG	HQ668089.1
*CAT* R	TTT​CCC​TCA​GCA​TTG​ACC​AG	​
*GSH-PX* F	AGG​GAA​GGT​GAT​TCT​TGT​GGA	FJ670566.1
*GSH-PX* R	TTA​CAG​GGG​AAA​GCC​AGG​A	​
*SOD* F	GTG​GCT​GGG​ACA​ATC​GTT​T	DQ121374.1
*SOD* R	GTC​TTA​TTT​CGG​CAT​CAG​GC	​
*ACP* F	GAT​GAG​GGA​TTC​AAG​CCC​AGT​T	KT765023.1
*ACP* R	CTT​TGT​GCA​TGA​ACA​TGA​CCC​TG	​
*HSP70* F	TGA​CAA​GGG​TCG​CCT​CAG​TA	Liu et al. (2010)
*HSP70* R	CAT​TAT​CTT​GTT​GCG​ATC​CTC	​
*TOLL* F	TTC​GTG​ACT​TGT​CGG​CTC​TC	This study
*TOLL* R	GCA​GTT​GTT​GAA​GGC​ATC​GG	​
*RXR* F	GAT​CGG​CAG​TCC​CCT​TTG​AA	MZ501612.1
*RXR* R	TTG​GAC​ACA​CTG​GGA​GAA​GC	​
*ECR* F	ACA​GTT​CAG​CTC​ATA​GTG​GA	Li et al. (2025a)
*ECR* R	CTC​TCA​GCA​TCA​TCA​CTT​CG	​

### 2.6 Analysis of gut microbiota diversity

After 28 days of exposure, intestinal contents were collected from 30 shrimp in each group (6 individuals per sample, 5 replicates). Total DNA was extracted using the CTAB method. DNA quality was assessed by 1.0% agarose gel electrophoresis and a NanoDrop 2000 spectrophotometer. The V3–V4 hypervariable region of the bacterial 16S rRNA gene was amplified via PCR using primers 341F (5′-CCTACGGGNGGCWGCAG-3′) and 806R (5′-GGACTACHVGGGTATCTAAT-3′). The PCR reaction system (20 μL) contained 10 μL of 2×Taq Master Mix, 1 μL each of forward and reverse primers, 1 μL DNA template, and 7 μL ddH_2_O. The amplification program was as follows: initial denaturation at 95 °C for 3 min; 30 cycles of denaturation at 95 °C for 30 s, annealing at 55 °C for 30 s, and extension at 72 °C for 45 s; followed by a final extension at 72 °C for 10 min. Purified PCR products were used to construct an Illumina MiSeq sequencing library for high-throughput sequencing. Based on the obtained operational taxonomic unit (OTU) sequences and their abundance information, further analyses were conducted, including microbial species annotation, community structure analysis, indicator species identification, alpha-diversity assessment, and functional prediction of the microbiota (Liufu et al., 2025). The 16S rDNA high-throughput sequencing was performed by Guangzhou Genedenovo Biotechnology Co., Ltd.

### 2.7 Data processing and statistical analysis

The experimental data are presented as mean ± standard error of the mean (Mean ± SEM). All statistical analyses were performed using GraphPad Prism 9.3.1 software (GraphPad Software, San Diego, CA, United States). Prior to conducting one-way ANOVA, the Shapiro-Wilk test was used to assess the normality of each dataset (growth indicators, gene expression levels), and Levene’s test was employed to evaluate the homogeneity of variances among groups. The test results indicated that all data met the assumptions of normality (*p* > 0.05) and homogeneity of variances (*p* > 0.05). Subsequently, one-way ANOVA was used to compare overall differences among groups, followed by Tukey’s multiple comparison test for pairwise comparisons between groups. The significance level was set at *p* < 0.05.

## 3 Results

### 3.1 Effects of PVC-MPs on the survival rate and growth performance of *Macrobrachium rosenbergii*

**FIGURE 2:**
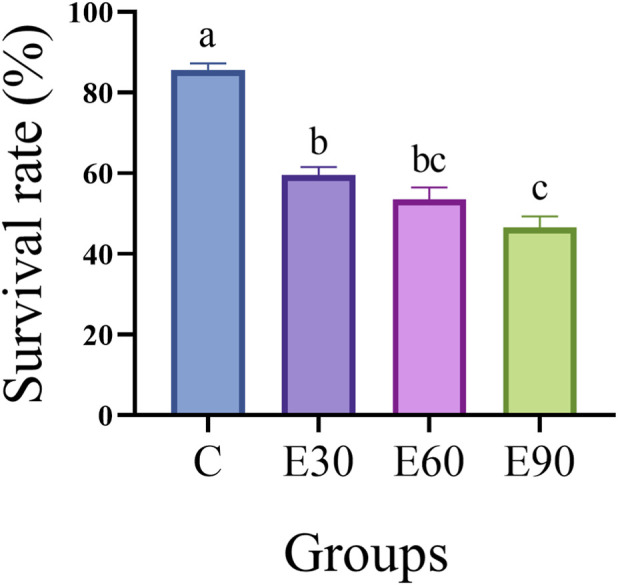
Effects of 28-day exposure to PVC microplastics with different particle sizes on the survival rate of *Macrobrachium rosenbergii.* Data are presented as mean ± standard error of the mean (SEM) (n = 4 replicate tanks per group, with 50 prawns per tank initially). Statistical analysis was performed using one-way ANOVA followed by Tukey’s multiple comparison test. Different lowercase letters indicate significant differences among groups (*p* < 0.05).

The results of the 28-day microplastic exposure experiment revealed that PVC-MPs significantly reduced the survival rate of *M*. *rosenbergii* (Figure 2). The survival rates in the E30, E60, and E90 groups were 59.50%, 53.50%, and 46.50%, respectively, all of which were significantly lower than that in the C group (85.5%, *P* < 0.05). Among the three experimental groups, the survival rate in the E90 group was significantly lower than that in the E30 group (*P* < 0.05). This indicates that PVC-MPs have a significant inhibitory effect on the survival rate of *M*. *rosenbergii*, and the survival rate decreases with increasing particle size.

Furthermore, exposure for 28 days also significantly inhibited the growth of *M*. *rosenbergii* post-larvae. The final body weight and final body length in the C group (1.80 ± 0.59 g, 5.99 ± 1.04 cm) were significantly higher than those in the E30, E60, and E90 groups (*P* < 0.05), while no significant differences in final body weight or body length were observed among the three experimental groups (*P* > 0.05). Concurrently, the weight gain rate and specific growth rate in the C group were 69.70% ± 46.74% and 1.73% ± 0.90%/d, respectively, both of which were significantly higher than those in the three experimental groups (*P* < 0.05, Table 2).

**TABLE 2:** Effects of exposure to polyvinyl chloride (PVC) microplastics with different particle sizes on growth indices of *Macrobrachium rosenbergii* after 28 days.

Items	C	E30	E60	E90
IMW (g)	1.12 ± 0.54	1.12 ± 0.56	1.12 ± 0.64	1.12 ± 0.44
FMW (g)	1.80 ± 0.59^a^	1.43 ± 0.66^b^	1.32 ± 0.56^b^	1.31 ± 0.47^b^
IBL (cm)	3.64 ± 0.58	3.64 ± 0.60	3.64 ± 0.61	3.64 ± 0.57
FBL (cm)	5.99 ± 1.04^a^	5.42 ± 0.83^b^	5.36 ± 0.75^b^	5.35 ± 0.72^b^
WGR (%)	67. 90 ± 46.74^a^	32.57 ± 24.35^b^	30.04 ± 22.22^b^	28.54 ± 22.98^b^
SGR (%/d)	1.73 ± 0.90^a^	0.95 ± 0.62^b^	0.89 ± 0.57^b^	0.84 ± 0.61^b^

Data are reported as the mean ± SE, of three replicates (n = 30). Mean values within a row with unlike superscript letters were significant different (p < 0.05). IMW: initial weight; FMW: final weight; IBL: initial body length; FBL: final body length; WGR: wight gain rate; SGR: special growth rate.

### 3.2 Distribution characteristics of PVC-MPs in the respiratory, digestive, and circulatory systems of *Macrobrachium rosenbergii*

**FIGURE 3:**
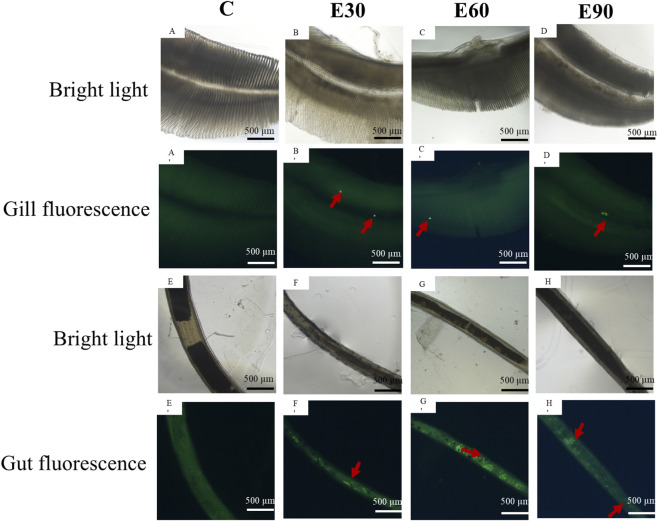
Accumulation characteristics of fluorescent PVC microplastics with different particle sizes in the intestines and gills of *Macrobrachium rosenbergii* during a 1-day exposure period. Note: Rows 1–2 show gill tissues, and rows 3–4 show intestinal tissues. Row 2 displays the corresponding white-light images of row 1. Columns 1–4 represent group C, group E30, group E60, and group E90, respectively. Scale bar = 500 μm.

The accumulation dynamics and spatial distribution characteristics of microplastics in key organs such as the gills and intestines of *M*. *rosenbergii* are shown in Figure 3. Fluorescent particles (indicated by red arrows) were detected in the gills and intestines of all three experimental groups as early as day 1 of exposure. In the gill tissues, only a small number of sporadically distributed fluorescent particles were observed in each experimental group. In contrast, the quantity of fluorescent microplastics in the intestinal tissues was significantly higher than that in the gills (*P* < 0.05). After 28 days of exposure to PVC-MPs, the quantitative analysis results of microplastics in the hemolymph are presented in Table 3. The concentrations of PVC-MPs in the hemolymph of post-larvae in the E30, E60, and E90 groups were 46.61 μg/g, 30.95 μg/g, and 67.83 μg/g, respectively. No microplastics were detected in the hemolymph of the control group (C). The results indicate that PVC-MPs rapidly accumulate in the gill and intestinal tissues of *M*. *rosenbergii* (with significantly higher levels in the intestine) and, after 28 days of exposure, can enter the hemolymph, thereby potentially adversely affecting the circulatory system of the juvenile prawns.

**TABLE 3:** Content of PVC microplastics with different particle sizes in the circulatory system of *Macrobrachium rosenbergii* after 28-day exposure (μg/g).

Groups	Content of microplastics in the samples
C	0
E30	46.61
E60	30.95
E90	67.83

### 3.3 Effects of PVC-MPs on the ultrastructure and apoptosis of *Macrobrachium rosenbergii*

**FIGURE 4:**
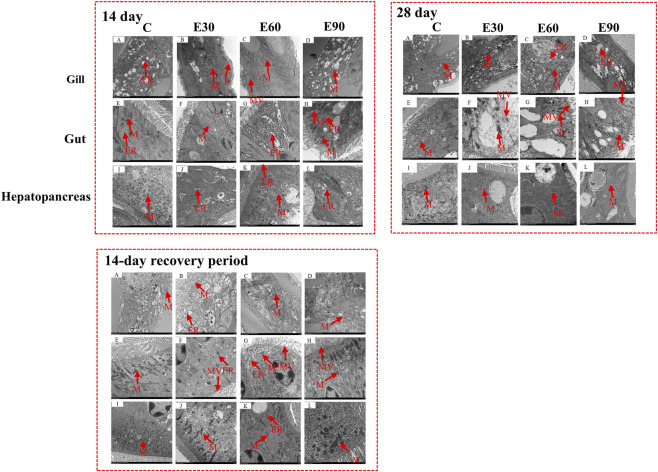
Ultrastructure of gill, intestinal and hepatopancreas tissues *of Macrobrachium rosenbergii* exposed to PVC microplastics with different particle sizes for 14 days, 28 days and after a 14-day recovery period (n = 3). In each panel, rows 1–3 represent gill, intestinal, and hepatopancreas tissues, respectively; columns 1–4 represent group C, group E30, group E60, and group E90, respectively. P: protuberance; ER: endoplasmic reticulum; M: mitochondrion; MV: microvilli. Scale bars = 2 μm.

The effects of microplastics on the subcellular structure of the gills, intestines, and hepatopancreas of *M*. *rosenbergii* are shown in Figure 4. After 14 and 28 days of exposure, significant edema was observed in the mitochondria (M) of gill filaments from all experimental groups, manifested as cristae disruption and matrix expansion. The rough endoplasmic reticulum (ER) was also markedly dilated and accompanied by vacuolization. Ultrastructural observation of the intestines revealed that after 14 days of exposure, the intestinal tissue of the experimental groups exhibited loose organization, luminal dilation, initial detachment of microvilli (MV), and vacuolization of mitochondria. By day 28 of exposure, the detachment of microvilli had intensified, and mitochondrial vacuolization became more pronounced. After 14 days of exposure, enlargement of intercellular spaces, vacuolization of mitochondria, cristolysis, and degranulation of rough endoplasmic reticulum in hepatocytes can be observed. These pathological changes become more severe after 28 days of exposure. After the 14-day recovery period, the morphology of the gill filaments showed a certain degree of recovery in all experimental groups. Mitochondrial vacuolization was partially alleviated, and the cristae structure gradually began to rebuild. In the intestinal tract, the length and density of the microvilli showed partial recovery, although localized vacuolization still persisted in the mitochondria and endoplasmic reticulum. In the hepatopancreas, the enlarged intercellular spaces were reduced, and cellular arrangement became more compact. The degree of mitochondrial vacuolization was alleviated, and the cristae structure was partially re-formed in some mitochondria. Transmission electron microscopy revealed that PVC-MPs caused progressive and tissue-specific ultrastructural damage to key organs (gills, intestines, and hepatopancreas) of *M*. *rosenbergii*. However, these subcellular structures exhibited a certain degree of reversible recovery after the cessation of exposure.

**FIGURE 5:**
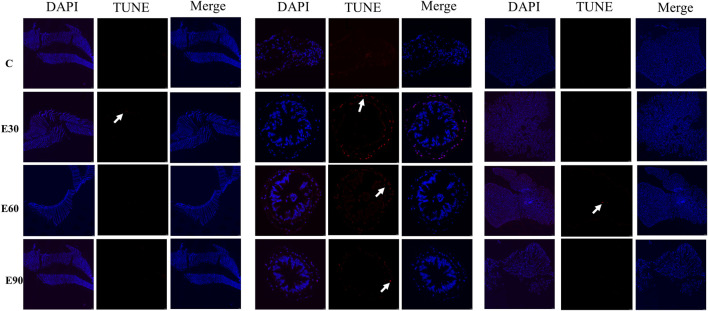
Apoptosis detection in the gill, intestine, and hepatopancreas of *Macrobrachium rosenbergii* after 28-day exposure under different particle size groups. Blue fluorescence indicates nuclei and red fluorescence indicates apoptotic cells (n = 3). Note: Row 1 shows gill, intestine, and hepatopancreas tissues of group C in order; Row 2 shows those of group E30; Row 3 shows those of group E60; Row 4 shows those of group E90.

To investigate the damage mechanisms of PVC-MPs on the tissue structure of *M*. *rosenbergii*, this study employed fluorescence microscopy to detect apoptotic activity in the gill, hepatopancreas, and intestinal tissues (Figure 5). After 28 days of exposure, no obvious TUNEL-positive signals were detected in the gill, intestinal, or hepatopancreatic tissues of the control group (Group C). In contrast, apoptotic signals were observed in the exposed groups. Among them, TUNEL-positive signals in the nuclei of gill tissues were particularly pronounced in the E30 group. Apoptosis analysis of the intestinal tissue revealed that the number of apoptotic cells in all experimental groups was significantly higher than that in the C group (*P* < 0.05), with the E30 group exhibiting the highest apoptosis rate. In the hepatopancreatic tissue, faint yet distinct apoptotic signals were also detected across all exposure groups. Therefore, exposure to PVC-MPs for 28 days induced apoptosis in the gills, intestine, and hepatopancreas of *M*. *rosenbergii*, with the most obvious apoptosis observed in the intestine and strong signals detected in the gills at low concentrations, suggesting that apoptosis is an important mechanism underlying tissue damage caused by microplastics.

### 3.4 Effects of PVC-MPs on related gene expression in *Macrobrachium rosenbergii*

**FIGURE 6:**
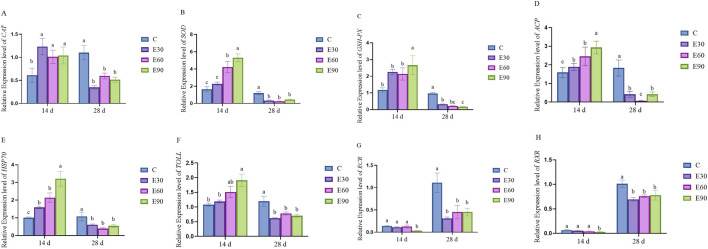
Effects of exposure to PVC microplastics with different particle sizes on related gene expression in *Macrobrachium rosenbergii* after 14 and 28 days (n = 3). **(A)** *CAT* gene expression levels in different treatment groups after 14 and 28 days of exposure; **(B)** *SOD* gene expression levels in different treatment groups after 14 and 28 days of exposure; **(C)** *GSH-PX* gene expression levels in different treatment groups after 14 and 28 days of exposure; **(D)** *ACP* gene expression levels in different treatment groups after 14 and 28 days of exposure; **(E)** *HSP70* gene expression levels in different treatment groups after 14 and 28 days of exposure; **(F)** *TOLL* gene expression levels in different treatment groups after 14 and 28 days of exposure; **(G)** *ECR* gene expression levels in different treatment groups after 14 and 28 days of exposure; **(H)** *RXR* gene expression levels in different treatment groups after 14 and 28 days of exposure. *CAT*: Catalase; *SOD*: Superoxide Dismutase; *GSH-PX*: Glutathione Peroxidase; *ACP*: Acid Phosphatase; *HSP70*: Heat Shock Protein 70; *TOLL*: ToLL; *ECR*: Retinoid X Receptor; *RXR*: Ecdysone Receptor. Data are presented as mean ± standard error of the mean (SEM), with three biological replicates per treatment group. Statistical analysis was performed using one-way ANOVA followed by Tukey’s multiple comparison test to analyze differences among different particle size groups at the same time point. Different lowercase letters indicate significant differences among groups at the same time point (*p* < 0.05).

The expression levels of antioxidant-, immune-, and growth-related genes in the hepatopancreas of *M*. *rosenbergii* after exposure to PVC-MPs are shown in Figure 6. Among the antioxidant-related genes (*CAT*, *SOD*, and *GSH-PX*), after 14 days of exposure, the expression levels of *CAT* and *SOD* in the E30, E60, and E90 groups were significantly higher than those in the C group (*P* < 0.05). Specifically, the *SOD* expression in the E60 group was significantly higher than that in the E30 group (*P* < 0.05), while the *GSH-PX* expression in the E90 group was significantly higher than that in the C, E30, and E60 groups (*P* < 0.05). After 28 days of exposure, the expression levels of *CAT*, *SOD*, and *GSH-PX* in the C group were significantly higher than those in the three experimental groups (*P* < 0.05). Moreover, the *GSH-PX* expression in the E30 group was significantly higher than that in the E90 group (*P* < 0.05). Regarding immune-related genes (*ACP*, *HSP70*, and *TOLL*), at 14 days, the expression levels of *ACP* and *HSP70* in the C group were highly significantly lower than those in the three experimental groups (*P* < 0.01), with the expression levels of these two genes in the E30 and E60 groups being significantly lower than those in the E90 group (*P* < 0.05). The expression of *TOLL* in the C and E30 groups was significantly lower than that in the E90 group (*P* < 0.05), while no significant difference was observed between the E60 and E90 groups (*P* > 0.05). After 28 days of exposure, the expression levels of the *ACP*, *HSP70*, and *TOLL* genes in group C were significantly higher than those in the three experimental groups (*P* < 0.05). Among growth-related genes (*ECR* and *RXR*), after 14 days of exposure, the expression levels of *ECR* and *RXR* in the E90 group were significantly higher than those in the C, E30, and E60 groups (*P* < 0.05). However, when the exposure period was extended to 28 days, the expression levels of *ECR* and *RXR* in the E30, E60, and E90 groups were significantly higher than those in the C group (*P* < 0.05). These results indicate that exposure to PVC-MPs initially activates (14 days) and subsequently inhibits (28 days) the expression of antioxidant and immune-related genes in the hepatopancreas of *M*. *rosenbergii*, while the growth-related genes are continuously and significantly upregulated with prolonged exposure. This suggests that under long-term stress, the organism’s defense capacity is impaired and normal growth may be disrupted.

### 3.5 Effects of PVC-MPs on the gut microbiota

The effects of PVC-MPs exposure on the alpha diversity indices of the gut microbiota in *M*. *rosenbergii* are shown in Table 4. The Sobs and Chao1 indices for the four groups (Group C, E30, E60, and E90) ranged from 264.67 to 341.00 and 296.28 to 370.13, respectively. Both the Sobs and Chao1 indices of the three experimental groups were significantly lower than those of Group C (*P* < 0.05). The sequencing coverage across all groups ranged from 99.90% to 99.93%, with no significant difference observed (*P* > 0.05).

**TABLE 4:** Effects of 28-day exposure PVC microplastics with different particle sizes on intestinal microbial α-diversity of *Macrobrachium rosenbergii.*

Diversity index	Groups			
	C	E30	E60	E90
Sobs	341.00 ± 54.95^a^	268.67 ± 37.43^b^	271.67 ± 34.39^b^	264.67 ± 33.29^b^
Chao1	370.13 ± 43.18^a^	298.89 ± 39.20^b^	313.06 ± 39.93^b^	296.28 ± 36.60^b^
Coverage (%)	99.91 ± 0.02	99.92 ± 0.01	99.90 ± 0.01	99.93 ± 0.02

**FIGURE 7:**
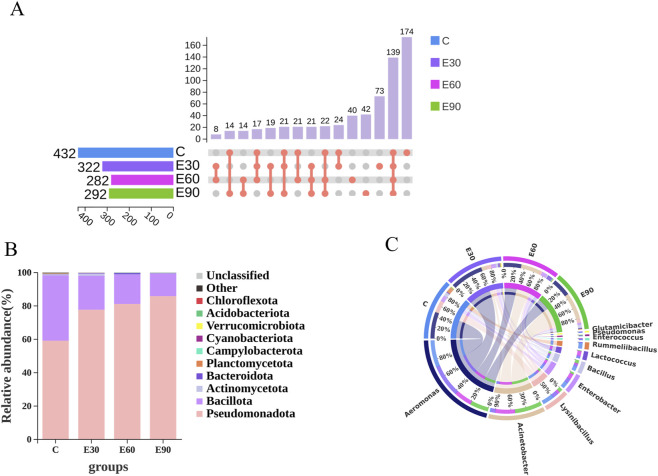
Effects of 28-day exposure to PVC microplastics of different particle sizes on the intestinal microbiota of *Macrobrachium rosenbergii*. **(A)** UpSet diagram of OTU numbers in different treatment groups; **(B)** Community composition of intestinal microbiota in different treatment groups at the phylum level (showing the top 12 taxa in abundance): bar plot; **(C)** Community composition of intestinal microbiota in different treatment groups at the genus level (showing the top 12 taxa in abundance): circos plot.Five biological replicates were set up for each group (n = 5), with each replicate consisting of pooled intestinal contents from six prawns. Data are presented as mean values. OTU: Operational Taxonomic Unit.

A total of 1,328 OTUs were obtained from the four groups, among which 139 were shared OTUs. Groups C, E30, E60, and E90 possessed 174, 73, 40, and 42 unique OTUs, respectively (Figure 7A). Relative abundance analysis at the phylum level revealed that the dominant phyla across all groups were Pseudomonadota, Bacillota, and Actinomycetota (Figure 7B). Compared to Group C, the abundance of Pseudomonadota significantly increased in Groups E30 and E60 (*P* < 0.05), and showed a highly significant increase in Group E90 (*P* < 0.01). At the Bacillota phylum level, Group C was significantly higher than Groups E30 and E60 (*P* < 0.05), and highly significantly higher than Group E90 (*P* < 0.01). Regarding Actinomycetota, no significant differences were observed among the four groups (*P* > 0.05). The compositional characteristics of the top ten most abundant bacterial genera in the intestine after exposure to PVC-MPs are shown in Figure 7C. The shared dominant genus across all groups was *Aeromonas*, with its abundance in the E30 group being significantly higher than that in the other three groups (*P* < 0.05). At the genus level of *Acinetobacter*, the E90 group was significantly higher than the E30 group (*P* < 0.05), highly significantly higher than the C group (*P* < 0.01), and showed no significant difference compared to the E60 group (*P* > 0.05). For *Lysinibacillus*, the C group was significantly higher than the E30 and E90 groups (*P* < 0.05) and highly significantly higher than the E60 group (*P* < 0.01). Regarding *Enterobacter*, there were no significant differences among the C, E30, and E60 groups (*P* > 0.05), all of which were significantly higher than the E90 group (*P* < 0.05). As for *Bacillus*, the C and E90 groups were significantly higher than the E30 group (*P* < 0.05) and highly significantly higher than the E60 group (*P* < 0.01). These results indicate that exposure to PVC-MPs significantly alters the diversity, structure, and composition of the intestinal microbial community in *M*. *rosenbergii*, leading to gut microbiota dysbiosis in juvenile prawns.

### 3.6 Residual characteristics of PVC-MPs in *Macrobrachium rosenbergii* during the recovery phase

**FIGURE 8:**
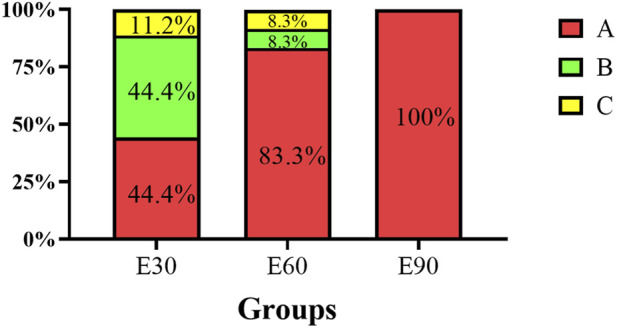
Proportion of fluorescently labeled PVC microplastics remaining in gill and intestinal tissues of *Macrobrachium rosenbergii* after 14 days of recovery. **(A)** No residual fluorescent microplastics were found in any of the two tissues. **(B)** Residual fluorescent microplastics in one tissue. **(C)** Residual fluorescent microplastics in two tissues. The number of individuals examined for each particle size group (E30, E60, E90) was n = 20, n = 20, and n = 20, respectively. Data are presented as percentages, representing the proportion of individuals within each group corresponding to each residual category. No error bars are shown as these are descriptive percentages from a single sampling.

After a 14-day recovery period, the residual status of PVC-MPs in the gills and intestines of *M*. *rosenbergii* was examined (Figure 8). The results indicate that clearance efficiency of PVC-MPs varied significantly among individuals. In the E30 group, 44.40% of individuals showed no detectable residual MPs, while 44.40% were found to have residues in a single tissue, and 11.20% had detectable residues in both the gills and intestinal tract. In the E60 group, 83.3% of the individuals completely cleared MPs from their bodies during the recovery period, while only 8.3% still retained residues in a single tissue or both tissues. In the E90 group, all individuals were able to completely eliminate the PVC-MPs from both their gills and intestinal tract.

## 4 Discussion

Exposure to PVC microplastics significantly inhibited the growth of juvenile *M. rosenbergii*, revealing a complex, size-dependent toxicity profile. Notably, organismal survival did not align with the degree of cellular damage. Large microplastics (90 μm) resulted in the highest mortality (46.50% survival; Figure 2) and the most severe growth restriction (Table 2), yet induced less tissue apoptosis than the 30 μm group (Figure 5). Despite exhibiting the highest tissue accumulation and most severe apoptotic response (Figure 5), prawns exposed to 30 μm particles maintained a comparatively higher survival rate (Table 2). These findings suggest that microplastic toxicity is not governed by a single factor—such as physical damage or oxidative stress—but rather operates through distinct, size-specific pathways.

Based on our findings, we propose a dual-toxicity model to explain these size-dependent mechanisms: large microplastics induce acute mortality via physical obstruction, whereas small microplastics drive chronic damage through bioaccumulation. Although 90 μm particles rarely penetrated tissues and were fully cleared during the depuration phase (Figure 8), their accumulation in the digestive tract and gill cavities severely restricted feeding and respiration. This physical blockade caused rapid energy depletion and respiratory failure, leading to mortality before significant cellular damage, such as apoptosis, could develop. This aligns with Kim et al. (2025), who reported that large microplastics in *Litopenaeus vannamei* cause mortality primarily through mechanical obstruction rather than cytotoxicity.

By contrast, 30 μm particles resulted in lower acute mortality but readily breached intestinal and branchial epithelial barriers to enter systemic circulation. Internal retention was high, with 44.4% of individuals still harboring particles post-depuration (Figure 8). Prolonged exposure to these embedded particles triggered sustained oxidative stress, promoted apoptosis, and progressively compromised the prawns’ immune defenses. This model clarifies distinct risk profiles for aquaculture and ecosystems. Large microplastics pose an immediate threat to prawn survival and farming yields. Meanwhile, the sublethal persistence of small microplastics facilitates their transfer up the food chain, presenting a more insidious, long-term environmental hazard.

Physical damage and tissue barrier disruption are the primary drivers of early microplastic toxicity. Once ingested, these particles adhere to gill filaments and accumulate within the intestinal lumen, directly compromising epithelial integrity (Sun et al., 2019). As gills serve as the main interface with the aquatic environment, such structural impairment severely disrupts physiological regulation (Zhang et al., 2025; Henry et al., 2012). We detected microplastics in the gills and intestines within the first day of exposure (Figure 3). Subsequent ultrastructural analysis revealed pronounced subcellular damage, including mitochondrial swelling, cristae disruption, and endoplasmic reticulum vacuolization (Figure 4). The 30 μm particles exhibited a higher capacity to breach these epithelial barriers, a finding consistent with their prolonged retention during the depuration phase. Within the intestine, epithelial degradation and the loss of microvilli diminish nutrient absorption and barrier function (Huang et al., 2020; Li et al., 2024), likely driving the significant growth retardation observed across all treated groups. Therefore, while large microplastics impair growth primarily through mechanical obstruction, smaller particles do so by penetrating tissues and inducing deep structural damage. Although the macroscopic outcome is identical, the underlying pathways are fundamentally distinct.

Chronic microplastic toxicity is fundamentally driven by oxidative stress and immune exhaustion. The gene expression analysis in this study showed that antioxidant (*CAT*, *SOD*, *GSH-PX*) and immune-related genes (*ACP*, *HSP70*, *TOLL*) followed a distinct biphasic trajectory. At day 14, significant upregulation indicated an active compensatory defense response (Figure 6); however, a sharp decline by day 28 suggested that prolonged exposure ultimately overwhelmed these physiological defenses, resulting in immune exhaustion. Despite this systemic suppression, the E30 group maintained elevated *GSH-PX* expression through day 28, which likely contributed to their higher survival rate relative to the E90 group. Simultaneously, the E30 prawns displayed the most severe apoptotic signals across the gills, intestine, and hepatopancreas (Figure 5). The prolonged tissue retention of these 30 μm particles continuously activates programmed cell death pathways. This mechanism aligns with findings by Yang et al. (2020) in goldfish, where small microplastics exceeded cellular tolerance thresholds to trigger widespread apoptosis.

Beyond direct tissue damage, microplastics also exerted indirect toxicity by destabilizing the gut microbiome. Exposure significantly reduced intestinal α-diversity (Sobs and Chao1 indices; Table 3) and altered the overall community structure. At the phylum level, all treatments depleted *Bacillota* and enriched *Pseudomonadota* (Figure 6A), a recognized hallmark of intestinal inflammation (Shin et al., 2015). Although the E90 group exhibited minimal tissue apoptosis (Figure 5), it sustained the most extreme microbial dysbiosis, marked by the sharpest spike in *Proteobacteria*. This profound microbiome disruption likely underpins the high mortality specific to this group. Furthermore, given the established link between microbial shifts and developmental retardation (Li et al., 2021), the widespread growth inhibition across all exposed prawns appears to be a direct secondary effect of dysbiosis-induced inflammation.

The translocation and depuration of microplastics govern the persistence and reversibility of their toxic effects. In this study, microplastics of all three sizes were detected in the hemolymph of *M. rosenbergii* (Table 3), demonstrating their capacity for systemic translocation across biological barriers. This aligns with observations in the Sydney rock oyster, *Saccostrea glomerata* (Scanes et al., 2019). Given that particles appeared in the gills and intestines by day 1 (Figure 3) alongside evident tissue damage (Figure 4; 5), we hypothesize that microplastics infiltrate the circulatory system by compromising epithelial integrity, subsequently inducing systemic toxicity in distal organs. Depuration efficiency was strictly size-dependent (Figure 8). While the 90 μm particles were completely cleared within 14 days, residues persisted in some individuals from the 30 μm and 60 μm groups. This corroborates patterns observed in *Eriocheir sinensis* and *Procambarus clarkii*, where smaller particles exhibit prolonged retention (Zhang X. et al., 2022; Han et al., 2023). Notably, 11.2% of the 30 μm group retained microplastics in both the gills and intestines post-recovery (Figure 8), suggesting that smaller particles pose a more enduring ecotoxicological risk. These results support the findings of Kim et al. (2018) and Watts et al. (2014), confirming that while depuration is co-regulated by species and tissue types, particle size remains a decisive factor.

While this study clarifies how PVC particle size influences toxicity, several limitations warrant further investigation. First, although our model identifies physical obstruction and bioaccumulation as primary drivers, we did not perform real-time monitoring of physiological indicators. Future research should integrate behavioral tracking to quantify the immediate impacts of different plastic sizes on feeding and respiratory rates. Second, this study assessed microplastics in isolation. In natural aquatic environments, these particles often coexist with heavy metals and organic pollutants; therefore, the combined toxicity and potential synergistic effects of such mixtures require systematic study. Finally, our 14-day recovery period captured only short-term depuration kinetics. The long-term persistence of small microplastics and their potential transgenerational effects remain unknown, necessitating extended studies over broader timescales to fully assess their ecological risk.

## 5 Conclusion

PVC-MPs inhibit the growth and development of *M. rosenbergii* through a combination of physical damage, oxidative stress, immune suppression, and gut microbiota dysbiosis. These toxic effects are strictly size-dependent: large microplastics trigger acute mortality via mechanical obstruction, whereas small microplastics induce chronic toxicity through prolonged tissue retention and biochemical interference. Ultimately, clarifying these distinct pathways provides a critical framework for accurately assessing and mitigating microplastic risks within freshwater aquaculture ecosystems.

## Data Availability

The original contributions presented in the study are included in the article/Supplementary Material, further inquiries can be directed to the corresponding authors.

## Supplementary material

SUPPLEMENTARY FIGURE S1 The composition and abundance distribution of microplastics in M. rosenbergii farming ponds in Zhaoqing and Zhuhai within the Pearl River Delta region.

SUPPLEMENTARY FIGURE S2 GC-MS spectra of PVC microplastics in the hemolymph of juvenile shrimp. Note: Red, black, pink, and blue lines represent the Con, E30, E60, and E90 groups, respectively.
