# Supplementary figures and images for: Effects of polyvinyl chloride microplastics with different particle sizes on growth, physiology, and intestinal microbiota of Macrobrachium rosenbergii
Source: Front Toxicol. 2026 Apr 10;8:1797231. doi: 10.3389/ftox.2026.1797231 (PMC13105459; doi:10.3389/ftox.2026.1797231)

## Slide 1
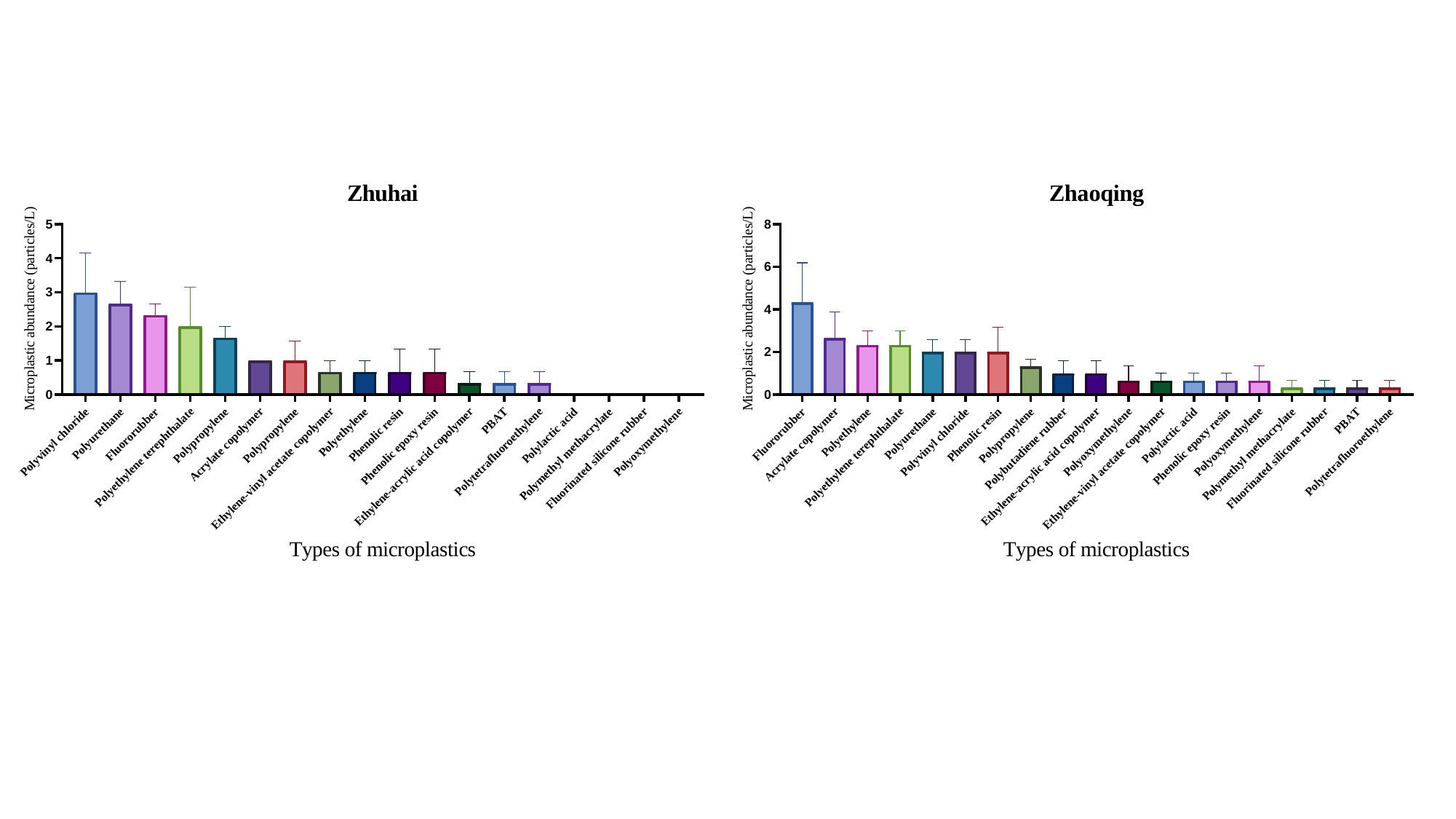

## Slide 2
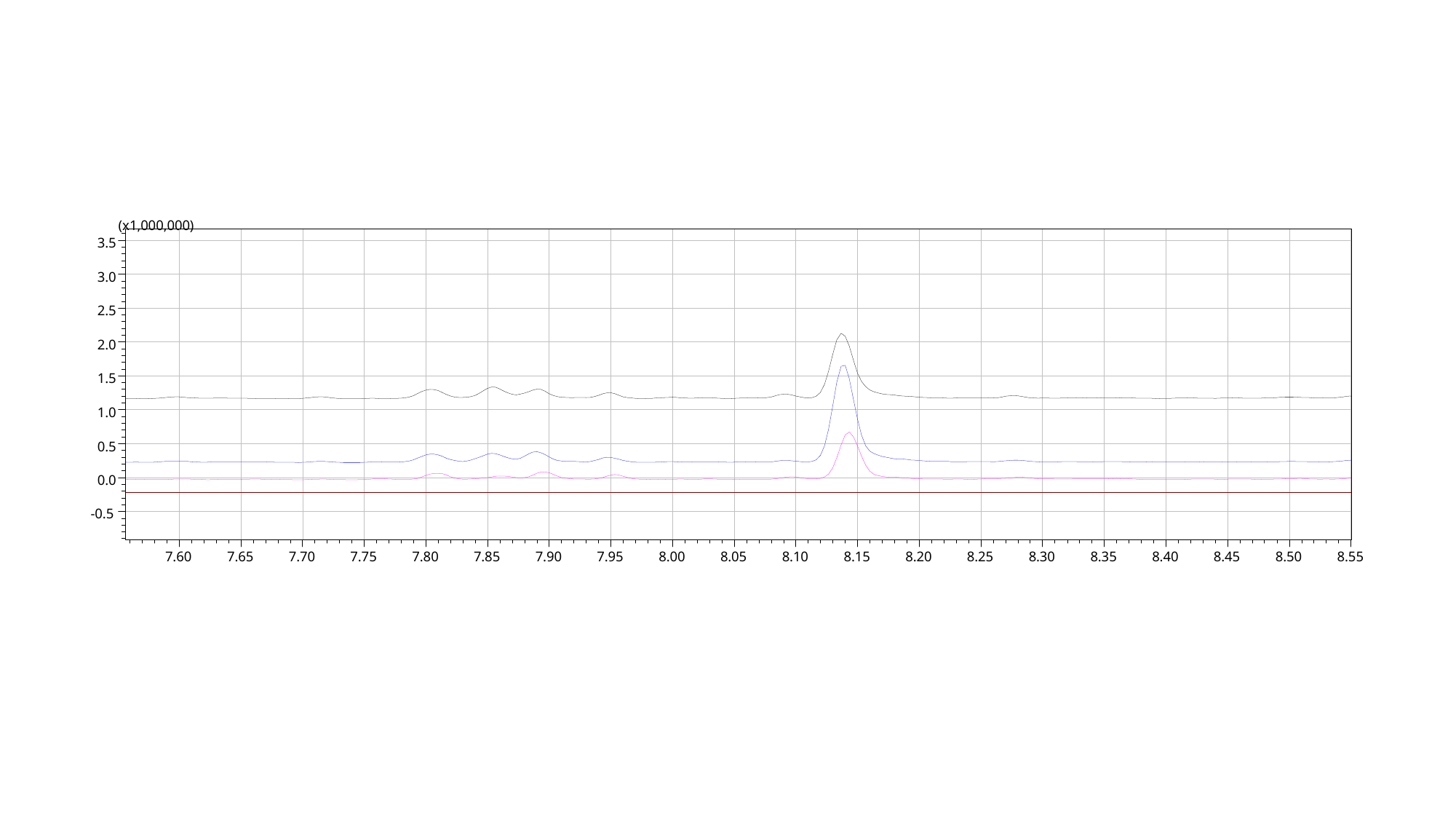

Supplement: Supplementary file 1 [file Presentation1.pptx]
